# Genome-wide identification and expression profiling of the SBP/miR156 module in response to pathogen stress in sisal (*Agave sisalana*)

**DOI:** 10.3389/fpls.2026.1781847

**Published:** 2026-03-20

**Authors:** Zhiwei Lu, Zhi Ke, Yanmei Zhang, Huibang Shen, Xiaowan Hou, Ziping Yang

**Affiliations:** 1 Zhanjiang Key Laboratory of Tropical Crop Genetic Improvement, South Subtropical Crops Research Institute, Chinese Academy of Tropical Agricultural Sciences, Zhanjiang, Guangdong, China; 2 Key Laboratory of Postharvest Physiology and Technology of Tropical Horticultural Products of Hainan Province, South Subtropical Crops Research Institute, Chinese Academy of Tropical Agricultural Sciences, Zhanjiang, Guangdong, China

**Keywords:** *Agave*, AhSBP, disease resistance, genome-wide identification, transcription factors

## Abstract

Sisal (*Agave sisalana*) productivity is severely threatened by purple leaf roll and zebra stripe diseases. The SQUAMOSA PROMOTER BINDING PROTEIN (SBP)-box gene family is an important regulator of plant defense against biotic stresses, yet its role in sisal immunity remains poorly understood. We performed a genome-wide identification of *AhSBP* genes in the *A.* sisalana genome and analyzed their expansion mechanisms through phylogenetic and synteny modeling. Comparative transcriptomics, promoter cis-element analysis, and RT-qPCR validation were further integrated to elucidate the regulatory mechanisms of the miR156-AhSBP module under pathogen stress. A total of 45 *AhSBP* genes were identified, revealing a lineage-specific expansion mainly driven by segmental duplication (70%). Among these, 18 *AhSBP* genes were identified as putative miR156 targets. Transcriptomic and qPCR analyses showed that asi-miR156 is significantly induced in susceptible cultivars, leading to the suppression of its direct targets, such as *AhSBP9* and *AhSBP38*. In contrast, resistant cultivars maintain low miR156 levels, allowing the activation of the SBP-mediated defense program. Our findings suggest that the miR156-AhSBP module serves as a primary regulatory hub coordinating sisal's immune homeostasis, while members like *AhSBP17* and *AhSBP34* likely function as downstream effectors. This study provides a comprehensive understanding of the *AhSBP* gene family and offers potential genetic targets for engineering broad-spectrum disease resistance in sisal.

## Introduction

Sisal (*Agave sisalana*. hybrid 11648) stands as one of the most economically significant natural fiber crops, globally across tropical and subtropical regions (Ferraz-Almeida and Ribeiro Ferreira da Silva, 2025). This hardy xerophytic plant produces strong, durable, and biodegradable fibers that find extensive applications in cordage, textiles, geotextiles, and composite materials (Gebretsadik et al., 2023). Beyond its fiber utility, sisal has gained increasing attention for its pharmaceutical potential, as recent studies have identified valuable secondary metabolites with anti-inflammatory and anticancer properties in its leaves (Costa et al., 2023). Moreover, the plant’s high biomass productivity and drought tolerance position it as a promising candidate for biofuel production in arid regions (Cushman et al., 2015). However, the global sisal industry faces mounting threats from devastating phytopathogens, particularly the rapidly spreading purple leaf curl disease and zebra stripe disease, which collectively account for annual yield losses exceeding 30% in major producing countries (S. Roy et al., 2011; Lu et al., 2023). These diseases not only reduce fiber yield by causing severe leaf necrosis and stunting but also compromise fiber quality through discoloration and reduced tensile strength (Raya et al., 2023). While conventional breeding efforts have made limited progress in developing resistant cultivars, molecular breeding represents a more potent alternative. However, the application of such modern techniques is significantly hindered by a poor understanding of sisal’s defense mechanisms, highlighting an urgent need for genomic-level investigations of potential resistance genes.

The SQUAMOSA PROMOTER BINDING PROTEIN (SBP)-box gene family represents a unique class of plant-specific transcription factors, including a highly conserved SBP domain with two zinc-binding sites (Cys2HisCys and Cys2His2) (Hou et al., 2017). Previous researches have shown that *SBP* genes exhibit specialized functional roles in plant development and stress responses (Gao et al., 2025). Besides, recent studies have also revealed that SBP play an important role in responding to pathogen attacks. For instance, AtSPL6 and NbSPL6 both exhibited an essential resistance against *Pseudomonas syringae* and Tobacco mosaic virus, respectively (Padmanabhan et al., 2013). Similarly, miR156/SPL9 module regulated the susceptibility to *Pseudomonas syringae* pv by affecting reactive oxygen species accumulation and immune response (Yin et al., 2019). These findings collectively position *SBP* genes as promising candidates for genetic improvement of disease resistance in crops. However, despite their demonstrated importance in plant-pathogen interactions, the *SBP* gene family has not been systematically investigated in sisal or other *Agave* spp species, representing a significant gap in our understanding of their potential roles in fiber crop defense mechanisms.

Current research on sisal molecular biology remains in its early stages compared to major crops, with only a small number of transcriptomic and genomic resources becoming available in recent years (Yang et al., 2024; López-Aguilar et al., 2021; Morreeuw et al., 2021). While these preliminary studies have identified several stress-responsive genes, a comprehensive analysis of disease resistance-related gene families, especially key transcription factors such as the SBP-box family, is currently lacking. This deficit severely constrains the development of molecular breeding strategies and the identification of genetic markers associated with disease resistance in sisal. Addressing these critical gaps requires systematic genome-wide identification of the *SBP* gene family in sisal, coupled with expression profiling under pathogen stress conditions.

To provide a foundation for sisal genetic improvement, we performed a multi-dimensional analysis of the *SBP-box* gene family in *A.sisalana*. We identified 45 *AhSBP* genes and explored their expansion mechanisms through phylogenetic modeling and Circos-based visualization of intra-genomic duplication events. Complementary analyses of gene structure, conserved motifs, and promoter cis-elements were employed to elucidate functional divergence. We also integrated comparative transcriptomics, promoter analysis, and RT-qPCR validation to analyze the regulatory mechanism mediated by miR156-AhSBP module against pathogen infection. This work constitutes the first systematic study of the *AhSBP* family, linking evolutionary patterns to potential functions in biotic stress resistance. The identified candidate genes offer valuable targets for future functional studies and breeding programs designed to combat mounting disease pressures.

## Materials and methods

### Plant materials and genome data acquisition

The complete genome sequence and annotation files for *A.sisalana.* hybrid 11648 (referred to as *Ah* for *A. sisalana.* hybrid 11648) were obtained from National Genomics Data Center under the project accession number PRJCA016359 (https://ngdc.cncb.ac.cn/gsub/). The corresponding protein sequences, coding sequences (CDS), and genomic sequences were downloaded for subsequent bioinformatics analyses. To facilitate functional validation and expression profiling, RNA-seq raw reads were utilized and have been deposited in the NCBI Sequence Read Archive (SRA) under BioProject PRJNA852945. Furthermore, the assembled mRNA sequences were deposited in GenBank with the following Submission IDs: 2723837, 2723846, 2723857, and 2723860.

### Identification and characterization of *AhSBP* genes

To comprehensively identify *SBP-box* gene family members in *A. sisalana*, two approach were employed. Firstly, known full-length SBP-box protein sequences from *Arabidopsis thaliana* (Retrieved from TAIR, www.arabidopsis.org) and *Oryza sativa* (From RAP-DB, rapdb.dna.affrc.go.jp) were used as query sequences in a local BLASTP (v2.12.0+) search against the *A. sisalana* protein database. An E-value threshold of 1e-5 was applied. Secondly, the Hidden Markov Model (HMM) profile corresponding to the SBP domain (Pfam: PF03110) was downloaded from the Pfam database (www.xfam.org) and utilized to scan the *A. sisalana* proteome using HMMER v3.3.2. All candidate sequences were then subjected to domain verification using both the Simple Modular Architecture Research Tool (SMART, smart.embl-heidelberg.de) and the Conserved Domain Database (CDD, www.ncbi.nlm.nih.gov/Structure/bwrpsb/bwrpsb.cgi) to ensure the presence of canonical SBP domains. Redundant sequences were eliminated, and the remaining verified genes were designated as *AhSBP* genes. Physicochemical properties of AhSBP proteins, including amino acid length, molecular weight, and theoretical isoelectric point (pI), were predicted using the ExPASy ProtParam tool (web.expasy.org/protparam/).

### Phylogenetic analysis and gene structure

The full-length amino acid sequences of all identified *AhSBP* genes, along with representative SBP-box proteins from *A.thaliana* and *O.sativa* for contextualization, were aligned using MAFFT v7 (Katoh and Standley, 2013) with default parameters. An unrooted maximum likelihood (ML) phylogenetic tree was constructed using IQ-TREE v2.1.2 (Minh et al., 2020), employing 1000 bootstrap replicates and automatically selected best-fit substitution models. The resulting phylogenetic tree was visualized and annotated with iTOL v6 (Subramanian et al., 2019). Based on the tree topology and clustering patterns, *AhSBP* genes were categorized into distinct subgroups. Exon-intron structures of *AhSBP* genes were extracted from the *A. sisalana* genome annotation files and visualized using the Gene Structure Display Server (GSDS 2.0, https://gsds.gao-lab.org/).

### Conserved motifs and *cis*-regulatory element analysis

Conserved protein motifs within the AhSBP amino acid sequences were identified using MEME Suite v5.4.1 (meme-suite.org/tools/meme). The parameters were set to identify 10 motifs with optimum widths ranging from 10 to 50 amino acid residues, allowing for any number of repetitions. Functional annotations for these discovered motifs were performed via the InterProScan online database (www.ebi.ac.uk/interpro/). For *cis*-regulatory element analysis, the 2000 bp upstream promoter regions from the translational start codon (ATG) of each *AhSBP* gene were extracted from the *A. sisalana* genome. These promoter sequences were then submitted to the PlantCARE database (Lescot et al., 2002) to identify putative *cis*-acting elements, with particular attention to those associated with stress responses.

### Chromosomal localization, gene duplication, and synteny analysis

The chromosomal locations and distribution of *AhSBP* genes were determined based on the *A. sisalana* genome annotation and mapped visually using MG2C online software (http://mg2c.iask.in/mg2c_v2.0/). Gene duplication events were identified by evaluating sequence similarity and genomic proximity. Tandem duplicates were defined as *AhSBP* genes situated within a 100 kb region on the same chromosome, separated by no more than five non-*AhSBP* genes, and sharing greater than 70% sequence identity over more than 70% of their coding sequence length. Segmental duplication blocks within the *A. sisalana* genome, which represent large-scale syntenic regions, were identified using MCScanX v1.1 (Wang et al., 2012) with an E-value cutoff of 1e-10 and a minimum block size of five genes. The identified intra-genomic synteny blocks and duplicated *AhSBP* gene pairs were graphically displayed using Circos v0.69–9 software (Krzywinski et al., 2009). For each identified duplicated *AhSBP* gene pair, the rates of synonymous (Ks) and non-synonymous (Ka) substitutions were calculated using KaKs_Calculator 2.0 (Wang et al., 2010). The approximate divergence time (T) for these duplicated pairs was estimated using the formula: T = Ks/(2 × 6.1 × 10^-9^) × 10–^6^ Mya (Million years ago).

### Expression profiling of *AhSBP* genes under pathogen infection

To assess the transcriptional responses of *AhSBP* genes to biotic stress, publicly available RNA-seq datasets generated from *A. sisalana* plants infected with sisal purple leaf roll disease and zebra stripe disease pathogens (BioProject: PRJNA852945) were utilized. Raw RNA-seq reads were initially processed using Trimmomatic v0.39 (Bolger et al., 2014) to remove adapter sequences and low-quality reads. Clean reads were then aligned to the *A. sisalana* reference genome assembly using HISAT2 v2.2.1 (Kim et al., 2019). Gene expression levels were quantified as Fragments Per Kilobase of transcript per Million mapped reads (FPKM) using StringTie v2.1.4 (Pertea et al., 2015). Differential expression analysis between pathogen-infected and mock-inoculated control samples was performed using the DESeq2 R package package (v1.34.0) (Love et al., 2014), applying criteria of an adjusted p-value < 0.05 and an absolute log_2_ Fold Change ≥ 1. Heatmaps illustrating the expression profiles of differentially expressed *AhSBP* genes were generated with TBtools v2.373 (Chen et al., 2020).

### Identification of asi-miR156 in sisal

The mature sequence of asi-miR156 in sisal was identified using a homology-based search against genomic datasets, using miR156 sequences from *A.thaliana*, *O.sativa*, and *Asparagus officinalis* as queries.

The structural features of *AhSBP* genes (**Supplementary Table 1**), including CDS, SBP, and 3’UTRs domains, were identified via the genomic datasets and NCBI-CDD databases. miR156 target sites were predicted using the psRNATarget (2017 release) server (Expectation ≤ 4.0). The coordinates for all gene features and miR156 target sites were normalized relative to the translation initiation codon (ATG), with the ‘A’ of the ATG assigned as position +1. The final integrated visualization was generated using TBtools (Chen et al., 2020).

### Plant materials and pathogen inoculation

Three sisal cultivars with different disease resistance (For purple leaf roll disease, *A.sisalana* hybrid 11648 (Susceptible) and its natural mutants in the field (Resistant); For zebra stiff leaf disease, *A.sisalana* hybrid 11648 (Susceptible) and Rema NO.1 (Resistant)) were used in this study. For purple leaf roll disease, artificial infestation was performed by transferring approximately 80 live *Dysmicoccus neobrevipes* individuals onto the leaf surfaces of both cultivars. To prevent interference from extraneous pests, all experimental plants were isolated using insect-proof netting. Leaf tissues (tip regions) were harvested at 0 d (pre-infestation), 60 d, and 90 d post-inoculation (dpi). All sampled materials were immediately frozen in liquid nitrogen and stored at -80°C for subsequent RNA-seq (This step has been completed previously) and RT-qPCR analyses. For each treatment, four individual plants were used, and the entire inoculation experiment was conducted in three independent biological replicates.

For zebra stiff leaf disease, healthy sisal leaves were subjected to wound inoculation. The tender basal regions of the leaves were surface-sterilized with 75% ethanol, followed by multiple rinses with sterile deionized water. The two cultivars were treated with mechanical wounding followed by the application of *Phytophthora nicotianae* Breda. Post-inoculation, high-humidity microclimates were established by misting the leaf surfaces with sterile water and sealing the plants with transparent film. A consistent humidity level was maintained by misting twice daily throughout the duration of the experiment. Leaf samples were harvested at 0, 24, 36, and 48 hours post-inoculation (hpi). Each treatment group consisted of five plants, and three independent biological replicates were performed. For all experiments, sampled tissues from the plants within each replicate were pooled and immediately snap-frozen in liquid nitrogen and stored at -80°C for subsequent RNA-seq (This step has been completed previously) and RT-qPCR analyses.

### RNA extraction and cDNA synthesis

Total RNA was extracted from frozen sisal tissues using the Plant RNA Kit (Omega Bio-tek, USA) following the manufacturer’s instructions. The integrity and concentration of RNA were assessed by agarose gel electrophoresis and NanoDrop 2000 spectrophotometry (Thermo Fisher Scientific, USA). For miRNA quantification: 1 ug of total RNA was reverse-transcribed into cDNA using the miRNA-specific stem-loop primer and the PrimeScript™ RT Reagent Kit (Takara, Japan). For *AhSBP* gene quantification: First-strand cDNA was synthesized from 1 ug of total RNA using the HiScript III RT SuperMix with gDNA Wiper (Vazyme, China).

### RT-qPCR analysis of asi-miR156 and *AhSBP* genes

Quantitative RT-PCR (RT-qPCR) was performed on the QuantStudio 6 Flex Real-Time PCR System (Applied Biosystems, USA) using SYBR Green Master Mix. The expression of mature asi-miR156 and five representative *AhSBP* genes (*AhSBP9*, *17, 27*, *34*, *38*) was quantified. U6 snRNA was used as the internal control for asi-miR156 normalization. For *AhSBP* mRNA expression, actin were employed as stable internal reference genes. The relative primer sequences were listed in **Supplementary Table S2**. The relative expression levels were calculated using the 2^-ΔΔCt^ method.

### Statistical analysis

All statistical computations and data visualizations were primarily carried out using R v4.3.1 statistical software (R Core Team, 2023) and GraphPad Prism v9.0 (GraphPad Software, LLC, USA). Specific statistical tests applied are detailed within the respective figure legends.

## Results

### Genome-wide identification, chromosomal distribution, and molecular characterization of the *AhSBPs* gene family

To define the composition and molecular characteristics of the *SBP-box* gene family in sisal (*A.sisalana* hybrid 11648), we conducted a systematic screen of the genome to identify all putative members containing the conserved SBP domain. A comprehensive genome-wide survey identified a total of 45 *SBP-box* genes within the *A.sisalana* hybrid 11648 genome. These genes were systematically named from *AhSBP1* to *AhSBP45* according to their physical locations on the linkage groups (LGs) and contigs. The physicochemical properties of the AhSBP proteins displayed remarkable heterogeneity (**Table 1**). The length of the encoded proteins varied more than nine-fold, ranging from a minimum of 114 amino acids (AhSBP14) to a maximum of 1,034 amino acids (AhSBP42). Accordingly, the molecular weights spanned a broad range from 12.65 kDa to 114.11 kDa. This extensive variation implies a significant functional diversification among family members. Regarding the isoelectric point (pI), the values ranged from 5.97 (AhSBP4) to 9.85 (AhSBP43). Notably, 84.4% of the proteins (38 out of 45) possessed a pI greater than 7.0, indicating that AhSBP transcription factors are predominantly basic proteins. This characteristic is consistent with their potential localization and interaction with DNA within the nucleus. To further verify their localization, we performed an integrated subcellular localization prediction using DeepLoc 2.0 and WoLF PSORT (**Supplementary Table S3**). The results revealed that all 45 AhSBP proteins were all localized in the nucleus with high confidence and conserved nuclear localization signals (NLS). Collectively, these fundamental profiles reveal the extensive structural complexity of the AhSBP family, laying a material basis for their distinct evolutionary fates and functional specialization.

**Table 1:** Identification and physicochemical characteristics of the *AhSBPs* gene family members in *A.sisalana* hybrid 11648.

Gene ID	Gene name	Chromosome	Chromosome location		pI	Molecular weight/kDa	AA	ORF/bp	Exon
EVM0010019	AhSBP1	LG01	9621400	9630663	8.9	105.49	949	2850	4
EVM0044373	AhSBP2	LG01	12943056	12981490	9.09	39.64	371	1116	4
EVM0011000	AhSBP3	LG01	342676253	342687320	9.61	27.19	249	750	11
EVM0030023	AhSBP4	LG01	319534435	319549127	5.97	96.90	881	2646	10
EVM0030348	AhSBP5	LG01	8859246	8869701	6.75	75.30	670	2013	6
EVM0022130	AhSBP6	LG01	148698306	148711582	8.84	48.84	450	1353	14
EVM0014783	AhSBP7	LG01	35147429	35149439	8.74	40.70	377	1134	5
EVM0011966	AhSBP8	LG01	34384911	34386987	9.11	32.27	285	858	3
EVM0042052	AhSBP9	LG01	7376303	7383666	9.54	36.23	325	978	3
EVM0033433	AhSBP10	LG02	391445784	391446940	8.77	19.36	178	537	3
EVM0031365	AhSBP11	LG02	449848772	449851421	8.78	35.20	323	972	3
EVM0034835	AhSBP12	LG03	470520747	470522073	9.31	35.60	329	990	2
EVM0025862	AhSBP13	LG03	469632478	469633640	9.62	32.27	292	879	3
EVM0049338	AhSBP14	LG03	206078454	206079334	8.54	12.65	114	345	2
EVM0030054	AhSBP15	LG05	366946641	366996412	8.35	74.14	657	1974	11
EVM0007387	AhSBP16	LG05	141222494	141228411	8.89	71.52	636	1911	13
EVM0005362	AhSBP17	LG05	368679494	368711668	8.55	87.04	781	2346	13
EVM0042711	AhSBP18	LG05	138917639	138923536	8.7	108.25	985	2958	12
EVM0054576	AhSBP19	LG06	128842895	128856516	8.45	28.61	261	786	12
EVM0025327	AhSBP20	LG06	18481788	18483092	8.88	104.89	951	2856	3
EVM0058080	AhSBP21	LG06	6195450	6206746	8.85	52.33	481	1446	23
EVM0034745	AhSBP22	LG06	182330193	182332429	8.32	68.54	611	1836	5
EVM0053024	AhSBP23	LG06	28930722	28934575	9.15	34.09	310	933	10
EVM0056930	AhSBP24	LG07	78092296	78094112	9.45	35.93	326	981	3
EVM0052490	AhSBP25	LG07	77689464	77691504	9.5	28.14	252	759	3
EVM0021933	AhSBP26	LG09	36107652	36110385	9.08	20.14	181	546	4
EVM0049199	AhSBP27	LG09	35402517	35405070	9.08	20.14	181	546	4
EVM0056691	AhSBP28	LG14	1209504	1215909	6.52	43.70	403	1212	3
EVM0010119	AhSBP29	LG19	2606018	2617671	6.57	44.60	409	1230	3
EVM0048942	AhSBP30	LG20	68354761	68365065	8.79	36.93	337	1014	13
EVM0026773	AhSBP31	LG20	61268823	61270317	6.69	106.97	970	2913	2
EVM0033874	AhSBP32	LG20	46183416	46198822	9.6	33.08	297	894	3
EVM0025720	AhSBP33	LG25	64746084	64747736	9.8	20.04	183	552	2
EVM0033898	AhSBP34	LG25	63695797	63697569	8.69	23.26	214	645	2
EVM0047112	AhSBP35	LG26	16555118	16556601	8.71	17.59	159	480	3
EVM0014887	AhSBP36	LG27	17587657	17602250	8.18	111.15	1012	3039	11
EVM0046307	AhSBP37	LG28	38971297	38977147	7.02	43.89	399	1200	4
EVM0019822	AhSBP38	LG30	17108068	17121760	8.81	36.43	330	993	4
EVM0015658	AhSBP39	LG30	35223432	35238655	8.8	52.91	481	1446	3
EVM0006927	AhSBP40	Contig02035	724407	739879	9.41	30.52	281	846	11
EVM0030875	AhSBP41	Contig02072	273303	281999	8.49	105.13	941	2826	3
EVM0006899	AhSBP42	Contig02598	180075	184416	8.53	114.11	1034	3105	16
EVM0026927	AhSBP43	Contig03073	62416	93390	9.85	27.11	257	774	3
EVM0053578	AhSBP44	Contig05865	74138	78501	8.13	110.34	995	2988	13
EVM0011056	AhSBP45	Contig06097	19344	35463	6.65	89.27	796	2391	10

The *AhSBP* genes are listed according to their chromosomal positions. Gene ID, Unique identifier derived from the *A.sisalana* hybrid 11648 genome assembly. Location, Genomic coordinates (start and end positions) in base pairs (bp). pI, Theoretical isoelectric point. MW, Molecular weight in kilodaltons (kDa). AA, Length of the amino acid sequence. ORF, Length of the open reading frame in base pairs (bp). Exon, Number of exons in the coding sequence.

### Chromosomal distribution and genomic organization of *AhSBP* genes

**Figure 1:**
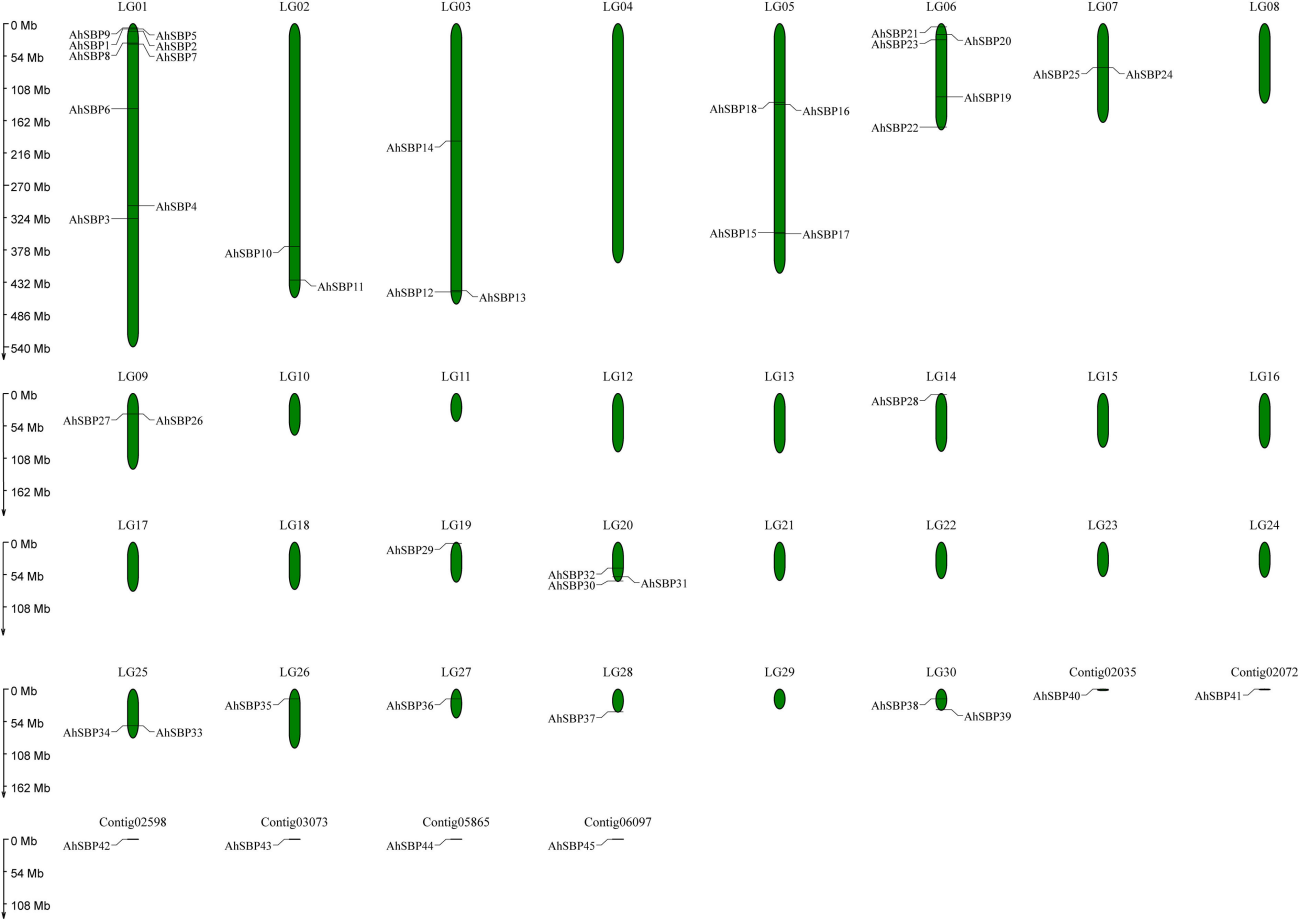
Chromosomal distribution of *AhSBP* genes in *A.sisalana* hybrid 11648. The chromosomal positions of the 45 *AhSBP* genes were mapped onto the sisal genome. The green bars represent the linkage groups (LGs) of the *A.sisalana* hybrid 11648 genome, among which 15 LGs were identified to harbor *AhSBP* genes. The scale on the left indicates the chromosomal length in megabases (Mb). Gene names are positioned on the right or left side of each chromosome corresponding to their physical locations.

To clarify the genomic distribution of the *AhSBP* family, the physical locations of all 45 identified *AhSBP* genes were mapped onto the *A.sisalana* hybrid 11648 genome (**Figure 1**). Among these, 39 genes were successfully anchored onto 15 specific linkage groups (LGs), while the remaining six genes (*AhSBP40*-*AhSBP45*) resided on unanchored contigs. LG01 was identified as the most gene-rich chromosome, harboring nine *AhSBP* members (approximately 20% of the total). A distinct ‘high-density island’ was observed on the upper arm of LG01 (0–54 Mb region), where six genes (*AhSBP1*, *AhSBP2*, *AhSBP5*, *AhSBP7*, *AhSBP8*, and *AhSBP9*) were tightly clustered. In contrast, the remaining genes on LG01 (*AhSBP3*, *AhSBP4*, and *AhSBP6*) were sparsely distributed along the middle and lower chromosome arms. Following LG01, LG06 contained five genes, and LG05 contained four genes. Interestingly, while the genes on LG06 also showed a tendency to cluster near the upper distal end (*AhSBP20*, *AhSBP21*, and *AhSBP23*), the four genes on LG05 were spaced relatively evenly across the chromosome. No *AhSBP* loci were detected on 15 linkage groups, including LG04, LG08, and LG10 to LG18 (excluding LG14), indicating that the family expansion occurred selectively in specific genomic regions rather than randomly across the entire genome. Furthermore, a prevalent telomeric or distal preference was observed in the gene distribution. A substantial proportion of *AhSBP* genes were located at the ends of the chromosomes. For instance, genes on LG02, LG03, LG07, LG14, and LG26 were predominantly positioned at the chromosomal distinct termini. In addition to the major cluster on LG01, smaller tandem arrays were evident on other linkage groups, including the tight triplet cluster of *AhSBP30*, *AhSBP31*, and *AhSBP32* on LG20, and the gene pair *AhSBP33*/*AhSBP34* on LG25. These physical proximities strongly suggest that tandem duplication events were a primary driving force in the expansion of the *AhSBP* family, particularly for the formation of gene clusters on LG01, LG06, and LG20. In summary, the genomic architecture of the *AhSBP* family is characterized by significant spatial clustering and distinct telomeric preferences, underscoring the critical role of localized duplication events in shaping its evolutionary trajectory in *A.sisalana* hybrid 11648.

### Phylogenetic analysis and evolutionary divergence of the *AhSBP* gene family

**Figure 2:**
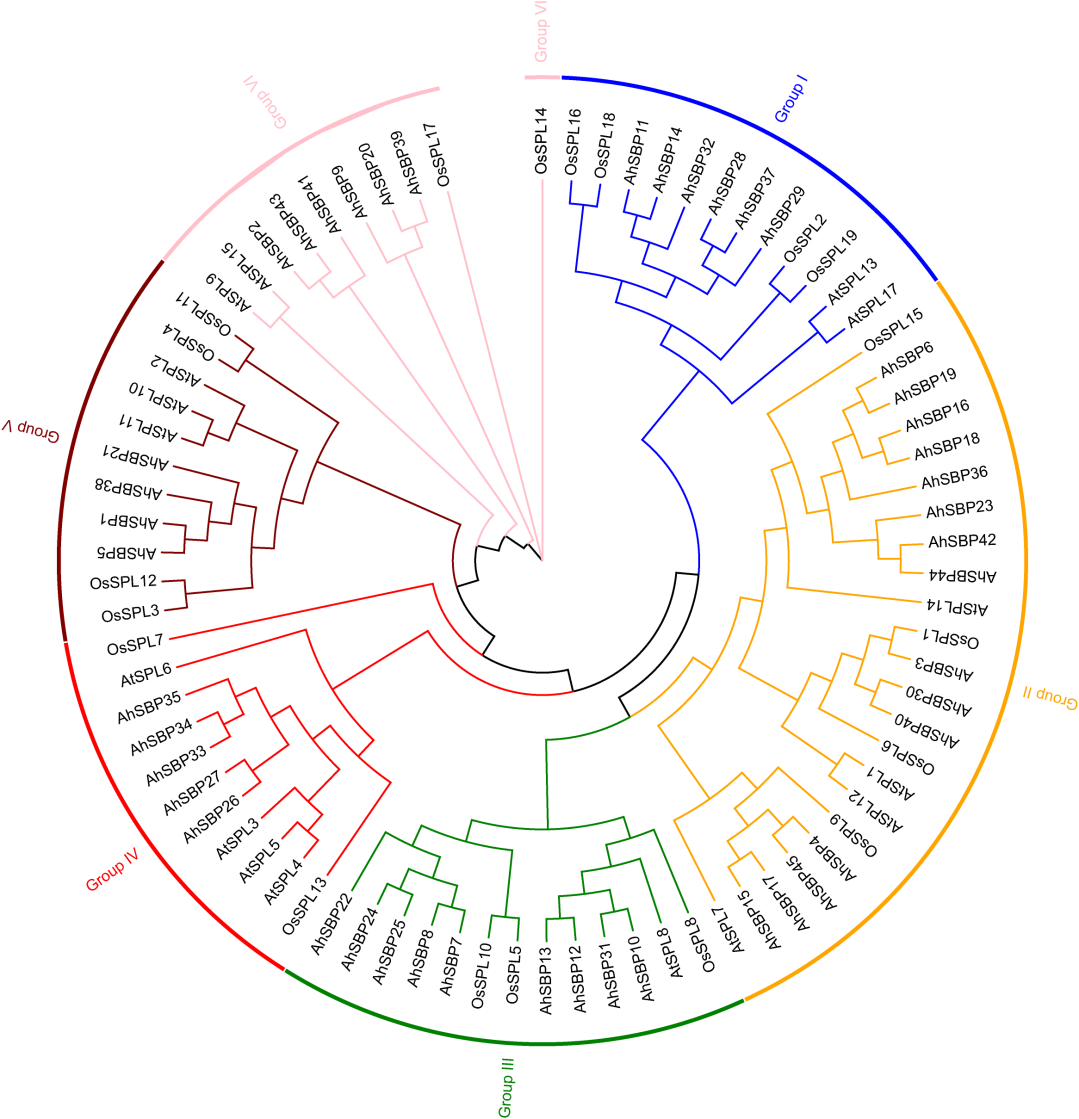
Phylogenetic analysis of SBP-box proteins from *A.sisalana* hybrid 11648, *A.thaliana*, and *O.sativa*. The unrooted phylogenetic tree was generated using the Maximum Likelihood (ML) method. The analysis included 45 AhSBPs, 16 AtSPLs, and 19 OsSPLs proteins. The proteins are clustered into six distinct groups (Groups I–VI), delineated by colored: Group I (blue), Group II (orange), Group III (green), Group IV (red), Group V (maroon), and Group VI (pink). Branches are colored according to their respective groups. The prefixes ‘Ah’, ‘At’, and ‘Os’ denote proteins from *A.sisalana* hybrid 11648 (sisal), *A.thaliana* (dicot reference), and *O.sativa* (monocot reference), respectively.

To elucidate the evolutionary history and orthologous relationships of the *AhSBP* family, a comprehensive phylogenetic tree was constructed using full-length protein sequences from *A.sisalana* hybrid 11648, *A.thaliana*, and *O.sativa* (**Supplementary Table S4**). Based on the topology and bootstrap support values, the SBP-box superfamily was classified into six distinct clades, designated as Groups I through VI (**Figure 2**). The *AhSBP* members were unevenly distributed among these subfamilies, revealing distinct evolutionary patterns. Group II constituted the largest subfamily, comprising 15 *AhSBP* genes. Within this clade, a striking lineage-specific expansion was observed. Specifically, a large subclade containing eight sisal genes (*AhSBP6, AhSBP16, AhSBP18, AhSBP19, AhSBP23, AhSBP36, AhSBP42, and AhSBP44*) clustered together without intervening *Arabidopsis* or rice sequences. This pattern strongly suggests that these genes originated from recent duplication events unique to the *A. sisalana* lineage. Similarly, Group III (9 members) and Group I (6 members) represented significantly expanded clades. In contrast, Group V was the smallest clade, containing only four members (*AhSBP1*, *AhSBP5*, *AhSBP21*, and *AhSBP38*), indicating a more conserved evolutionary history for this subgroup. Besides, phylogenetic affinity analysis revealed that AhSBP proteins generally exhibited a closer relationship with the monocot model *O.sativa* than with the dicot *A.thaliana*. In Group I, a distinct monocot-specific branch was identified, where six *AhSBP* genes (*AhSBP11, AhSBP14, AhSBP28, AhSBP29, AhSBP32*, and *AhSBP37*) formed a sister clade to the rice orthologs *OsSPL16* and *OsSPL18*. This topology reflects the divergence of monocots and dicots. Furthermore, specific orthologous relationships were evident in Group III, where *AhSBP10, AhSBP12, AhSBP13*, and *AhSBP31* clustered tightly with *OsSPL8* and *AtSPL8*. Conversely, the high degree of sequence divergence in Group IV and Group VI suggests that these members may have evolved novel functions specific to environmental adaptations in sisal. Taken together, these findings elucidate the evolutionary history of the *AhSBP* genes, characterized by strong monocot affinity and extensive recent duplications that have likely contributed to the functional plasticity of the SBP-box family in sisal.

### Gene structure and conserved motif composition of the *AhSBP* family

**Figure 3:**
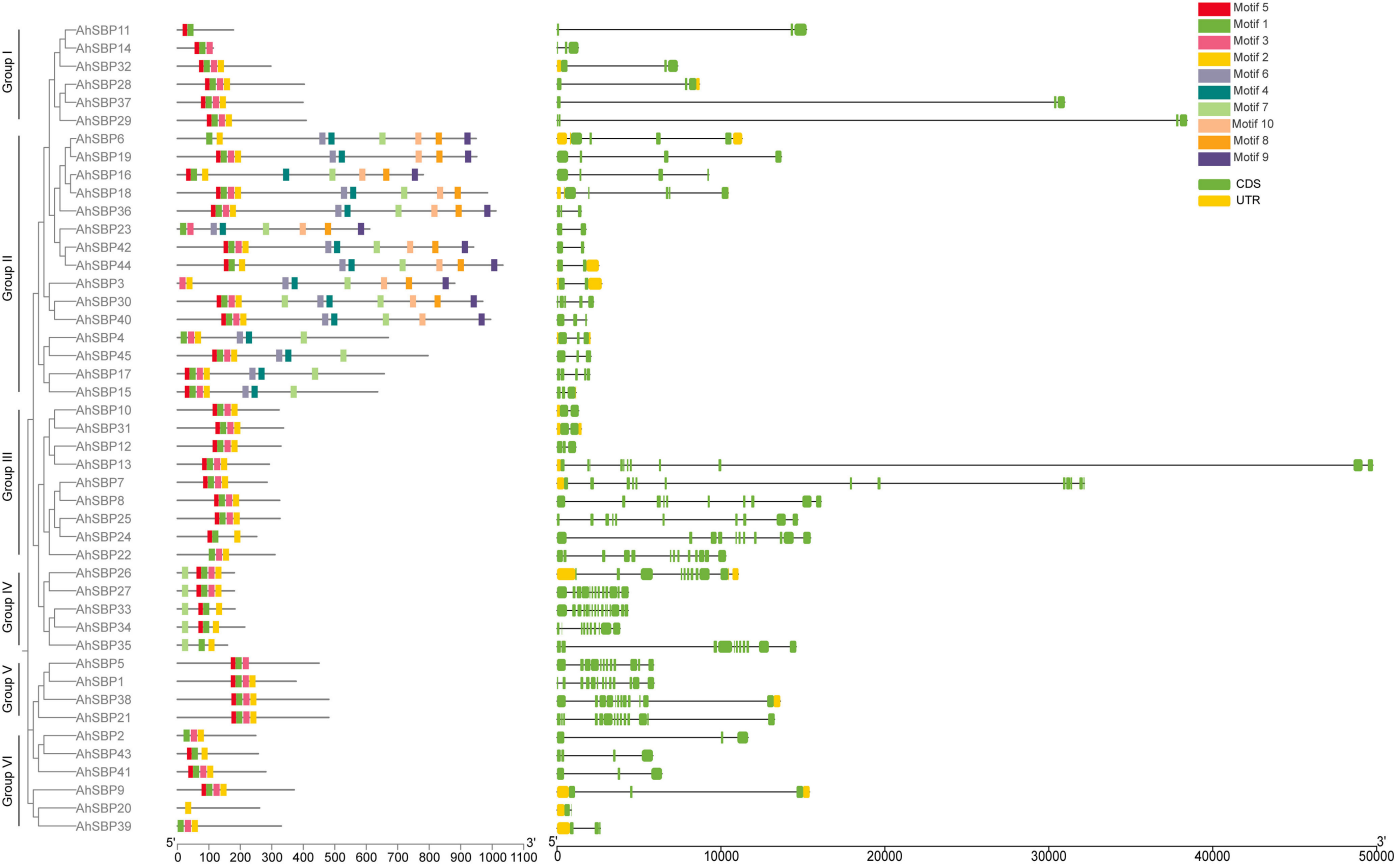
Conserved motif compositions and gene structural organizations of the *AhSBP* family. **(A)** Distribution of conserved motifs in AhSBP proteins. The ten predicted motifs are represented by different colored boxes, numbered 1–10 as indicated in the legend on the right. The length of the black line indicates the protein length. **(B)** Exon-intron structures of *AhSBP* genes. Green boxes, yellow boxes, and black lines represent coding sequences (CDS), untranslated regions (UTRs), and introns, respectively. The scale bar at the bottom indicates the length of the nucleotide sequences.

To investigate the structural diversity and potential functional conservation of AhSBP proteins, their conserved motifs and exon-intron structure were analyzed. A total of 10 conserved motifs from Motif 1 to Motif 10 were identified using the MEME suite (**Figure 3A**). The distribution of these motifs revealed a clear divergence in protein architecture among the six groups. Motifs 1, 2, and 5 were widely distributed across the majority of AhSBP members, likely representing the highly conserved SBP DNA-binding domain. Based on motif composition, the AhSBP family can be broadly categorized into two types. The first type comprises proteins with relatively short sequences (Including Group I/III/IV/V/VI), which primarily contain the core motifs (Motif 1, 2, and 5) but lack extensive C-terminal regions. The second type includes longer proteins (Including Group II) that exhibit a complex arrangement of additional motifs, such as Motif 4, 7, 8, 9, and 10. This specific combination of C-terminal motifs suggests that these members may possess distinct regulatory functions or activation domains compared to the shorter members.

The analysis of gene structure provided further insights into the genomic organization of the *AhSBP* family. As shown in the **Figure 3B**, the exon-intron structures exhibited remarkable variation. The number of exons ranged significantly, correlating with the protein length. While short genes typically contained 2–4 exons, the longer genes possessed highly fragmented structures with numerous exons (Such as *AhSBP44*). Significant variation in intron length was also observed. Several genes, such as *AhSBP2* and *AhSBP15*, contained long introns, which may harbor regulatory elements affecting gene expression. Taken together, these findings reveal that while the SBP domain remains highly conserved, significant variations in gene structure and motif composition have driven the structural divergence of the AhSBP family, potentially serving as the physical basis for their functional differentiation.

### Syntenic architecture and segmental duplication-driven expansion of the *AhSBP* family

**Figure 4:**
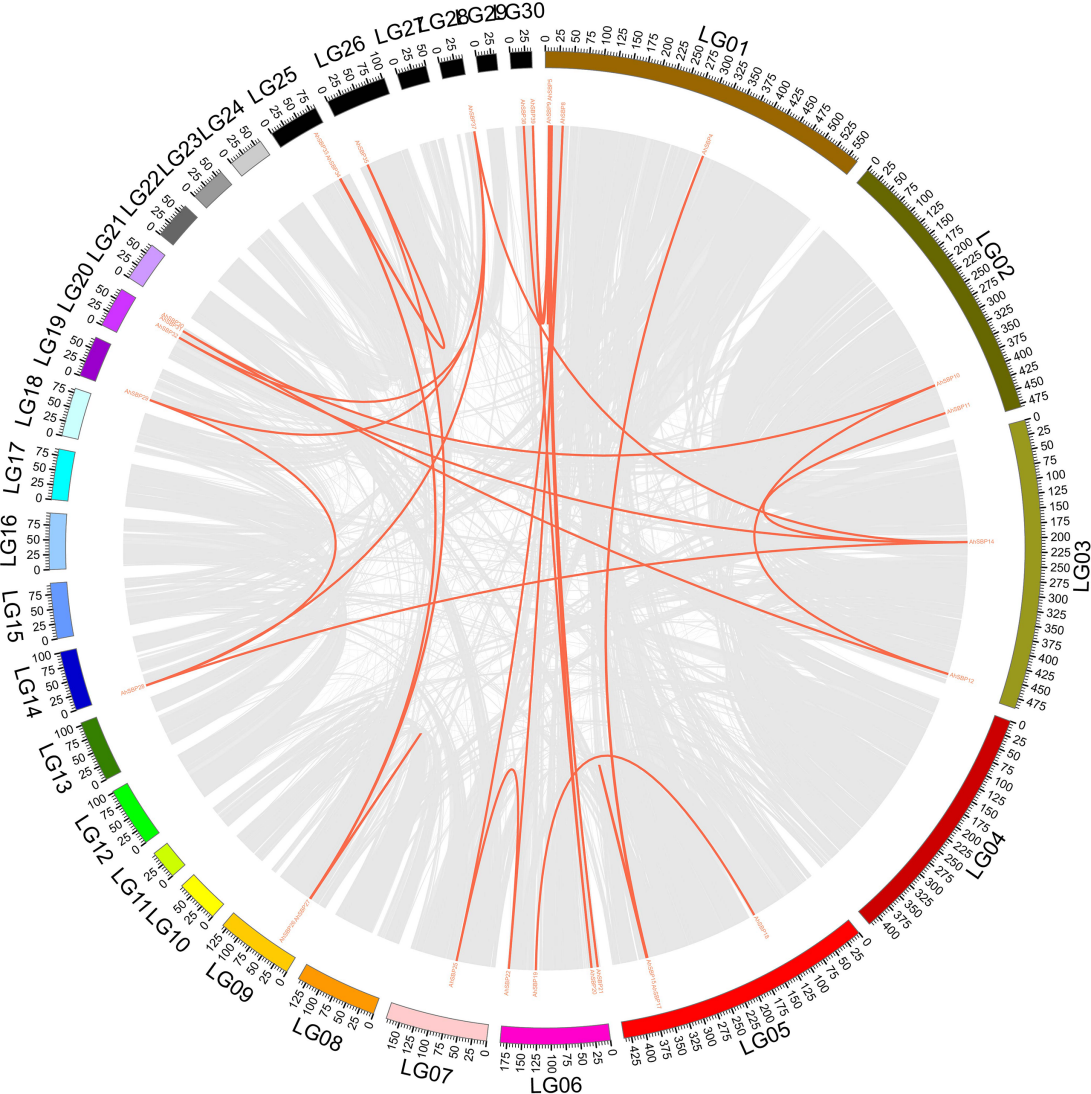
Genome-wide synteny and inter-chromosomal relationships of *AhSBP* genes. The Circos plot visualizes the genomic collinearity and 32 identified segmental duplication pairs. Outer Ring: The 30 linkage groups (LG01–LG30) of *A.sisalana* hybrid 11648 are color-coded, with physical distances marked in Mb. Red Links: Represent the 32 paralogous gene pairs originating from segmental duplication.

To decipher the evolutionary dynamics driving the expansion of the *AhSBP* family, we performed a genome-wide synteny analysis integrated with selection pressure (Ka/Ks) calculations (**Figure 4****;****Supplementary Table 5**). The results revealed that segmental duplication (including WGD-derived events) was the primary driver of family expansion, accounting for 32 paralogous pairs. These events gave rise to expansion hubs involving multiple chromosomes. A example is the regulatory linkage between LG01 (*AhSBP1*-*9*) and LG06 (*AhSBP19*-*23*). Similarly, LG03 (*AhSBP12*-*14*) served as a hub that connects to LG02, LG14, LG20, and LG28. Such cross-chromosomal connections suggested that gene family expansion is not limited to individual chromosomes but spreads widely across the genome. In addition to segmental events, tandem duplication significantly contributed to the local enrichment of *AhSBP* genes. Based on chromosomal proximity, two distinct tandem duplication clusters were confirmed: the *AhSBP33*/*AhSBP34* pair on LG25 and the *AhSBP38*/*AhSBP39* pair on LG30. These tandem duplications likely gave rise to recently evolved genes, enabling them to quickly take on specialized functions within certain linkage groups.

To evaluate the nature of selection pressure, Ka/Ks ratios were calculated for all 35 identified paralogous pairs. The results showed that all Ka/Ks ratios were less than 1.0 (ranging from 0.008 to 0.617), indicating that the *AhSBP* family has evolved under strong purifying selection to maintain the structural integrity of the SBP-box domain. However, varying selection intensities were observed between different duplication modes. For example, the tandemly duplicated pair *AhSBP33*/*AhSBP34* exhibited a Ka/Ks ratio of 0.681, suggesting a relatively strong selective constraint. This indicates that these tandem duplicates have largely maintained their ancestral functions to ensure a stable gene dosage effect within the same linkage group. Besides, segmental duplicates showed a broader range of divergence. For instance, the *AhSBP17*/*AhSBP15* pair (both located on LG05) and the *AhSBP38*/*AhSBP5* pair (LG30/LG01) exhibited relatively high ratios (0.617 and 0.351, respectively). While these values were still below 1.0, they represented a significant ‘relaxation of purifying selection’ compared to other members. In particular, the ratio of 0.617 for *AhSBP17*/*AhSBP15* was the highest in the family, signifying an accelerated evolutionary rate and substantial sequence divergence following segmental duplication. This increased genetic flexibility potentially allowed *AhSBP17* to undergo subfunctionalization or neofunctionalization, potentially enabling it to acquire specialized roles in sisal immunity and adaptation to biotic stress.

Collectively, our findings reveal a dual-mode expansion mechanism in the *AhSBP* family: Segmental duplications (responsible for bulk expansion) established inter-chromosomal regulatory networks, while tandem duplications (responsible for rapid innovation) drove functional specialization in confined genomic regions. Most *AhSBP* genes are under strong purifying selection to keep their SBP-domain functions integrity. This study provides a genomic framework for understanding how transcription factor families evolve and specialize in response to both genome-wide duplication events and local adaptive pressures.

### Characterization of Cis-acting regulatory elements in *AhSBP* promoters

**Figure 5:**
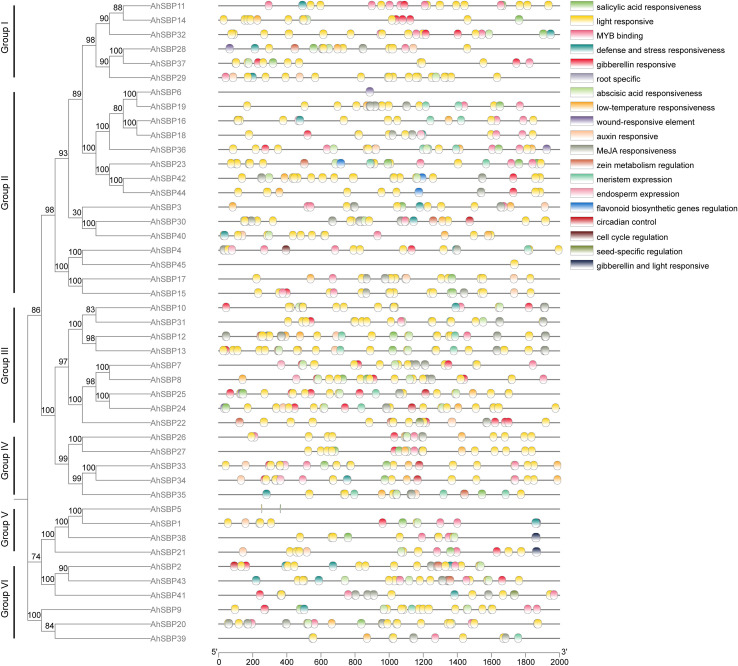
Predicted cis-acting regulatory elements in the promoter regions of *AhSBP* genes. The 2,000 bp upstream sequences from the translation start site (ATG) of *AhSBP* genes were analyzed. Left Panel: The distribution of cis-elements on the promoter sequences. The scale bar at the bottom indicates the distance from the transcription start site (bp). Right Panel (Legend): The different colored symbols represent specific categories of cis-acting elements. These include elements related to hormone responses (Abscisic acid, salicylic acid, auxin, gibberellin), abiotic/biotic stress (Light, low-temperature, defense, wound), and developmental processes (Such as meristem expression, zein metabolism, circadian control).

To elucidate the transcriptional regulatory mechanisms and potential functions of *AhSBP* genes, we identified cis-acting elements in the 2,000 bp upstream regions of all 45 AhSBP members (**Figure 5**; **Supplementary Table 6**). While light-responsive elements (such as G-box and Box 4) were ubiquitous, a detailed survey revealed that hormone- and stress-related motifs exhibit different distribution patterns, reflecting a high degree of functional specialization among different *AhSBP* members. Across the entire family, different members appeared to play specialized roles in plant growth and stress response. For instance, *AhSBP13* and *AhSBP16* belonging to Group II and Group III members respectively was characterized by motifs related to growth and development, including the meristem-expression element (CAT-box) and root-specific motifs. This suggested these members primarily orchestrate organogenesis and tissue differentiation. Some genes (Including *AhSBP24* and *AhSBP25*) in Group III harbored a high density of wound-responsive (WUN-motif) and low-temperature responsive (LTR) elements, indicating they are likely tuned to respond to physical damage and environmental cold stress. Crucially, a ‘defense-specialized cluster’ emerged. For example, the promoters of *AhSBP17* (Group II), *AhSBP27* (Group IV), *AhSBP9* and *AhSBP38* (Group V) standed out due to their enrichment of motifs linked to Systemic Acquired Resistance (SAR). Besides, *AhSBP38* and *AhSBP9* were also loaded with TC-rich repeats (defense and stress responsiveness) and TCA-elements (SA-responsiveness), suggesting they may be specifically equipped to integrate various biotic stress signals. Interestingly, *AhSBP34* displayed a contrasting profile; it was enriched in ABA-responsive (ABRE) and auxin-responsive motifs but lacked the dense SA/JA defensive clusters. This structural divergence implied that *AhSBP34* might prioritize ABA-mediated stress responses or growth-defense processes. In summary, the different roles of *AhSBP* genes appeared to be defined by their unique promoter architectures.

### Transcriptional dynamics and candidate gene discovery of *AhSBPs* under pathogen stress

**Figure 6:**
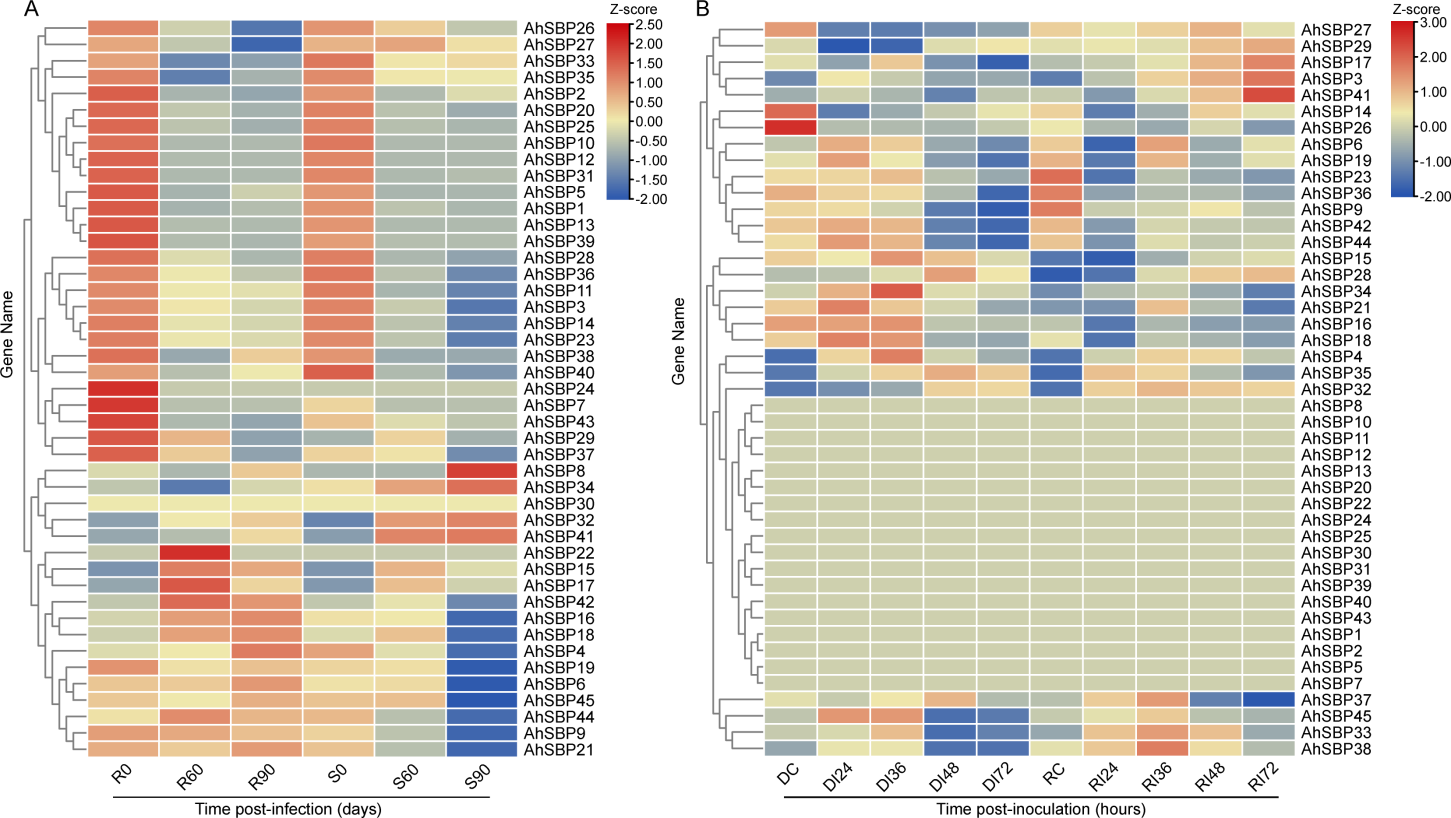
Comparative transcriptomic analysis of *AhSBP* genes under divergent pathogenic pressures. Heatmaps showing the relative expression levels (log2 FPKM) of *AhSBP* genes in resistant versus susceptible cultivars. The color scale shows the Z-score for each gene based on its log2-transformed FPKM values, with blue indicating low expression and red indicating high expression. **(A)** Transcriptional changes in resistant and susceptible cultivars during sisal purple leaf roll disease infection (0–90 days). R and S represent resistant and susceptible materials, respectively. The numbers 0, 60, and 90 refer to the sampling time points (days post-infection), where 0d serves as the uninfected control. **(B)** Transcriptional changes in resistant and susceptible cultivars during zebra stripe disease infection (24–72 hours). ‘D’ denotes the susceptible cultivar (Diseased) and ‘R’ denotes the resistant cultivar. ‘C’ and ‘I’ represent control and inoculated samples, respectively. The numbers 24, 36, 48, and 72 represent the sampling time points in hours post-inoculation (hpi). Data are presented as the mean of three independent biological replicates (n=3). Differential expression was determined using a threshold of |log_2_ (Fold Change)| ≥ 1.0 and an adjusted P-value <0.05.

To screen for potential transcriptional regulators associated with disease resistance in sisal, we performed a comparative transcriptomic analysis of the *AhSBP* family under two distinct pathological conditions. For sisal purple leaf roll disease (**Figure 6A**), the transcriptional profiles revealed a significant dichotomy between resistant (R) and susceptible (S) genotypes, reflecting fundamentally different adaptive strategies. A cluster of genes, including *AhSBP8*, *AhSBP32*, *AhSBP34* and *AhSBP41*, exhibited elevated expression specifically in the susceptible cultivar at late infection stages (S90). This pattern suggested these genes might be manipulated by the pathogen to facilitate infection or were associated with symptom development (Such as necrosis or leaf rolling). In contrast, another different group of genes, such as *AhSBP15*, *AhSBP16*, *AhSBP17*, *AhSBP18*, *AhSBP22*, and *AhSBP42*, demonstrated sustained upregulation in the resistant cultivar throughout infection period (R60–R90). In the susceptible line, these genes were suppressed. This implied that resistance to sisal purple leaf roll disease may rely on the sustained activation of specific *AhSBP* regulators, likely to maintain physiological homeostasis and suppress pathogen replication over long periods.

Unlike the long-term response to sisal purple leaf roll disease, the response to zebra stripe disease was characterized by rapid, acute transcriptional reprogramming within 72 hours. The heatmap results showed that some genes (*AhSBP 1/2/5/7/8/10/11/12/13/20/22/24/25/30/31/39/40/43*) showed negligible expression levels in leaf tissues (**Figure 6B**). Conversely, a substantial number of *AhSBP* genes exhibited robust transcriptional responses to the occurrence of zebra stripe disease. For instance, *AhSBP3/17/27/29/41* showed a clear induction pattern in the resistant line (R-I series) compared to the control, whereas *AhSBP4/5/16/18/21/28/34* showed up-regulated expression in the susceptible line. These genes potentially functioned as early responders, triggering the hypersensitive response (HR) or downstream defense pathways to restrict the pathogen immediately after invasion.

By integrating data from both diseases, we identified potential ‘broad-spectrum’ resistance candidates. Notably, *AhSBP17* appeared as a key positive regulator in both sisal purple leaf roll disease (high in R90) and zebra stripe disease (high in R-I lines). Conversely, *AhSBP34* was consistently associated with high expression in susceptible backgrounds in both datasets, marking it as a potential negative regulator or a susceptibility factor. Collectively, these distinct transcriptional signatures not only highlight the functional plasticity of *AhSBP* genes under biotic stress but also pinpoint *AhSBP17* and *AhSBP34* as high-priority targets for molecular breeding aimed at engineering broad-spectrum disease resistance in sisal.

### Identification of miR156 target sites in *AhSBP* genes

**Figure 7:**
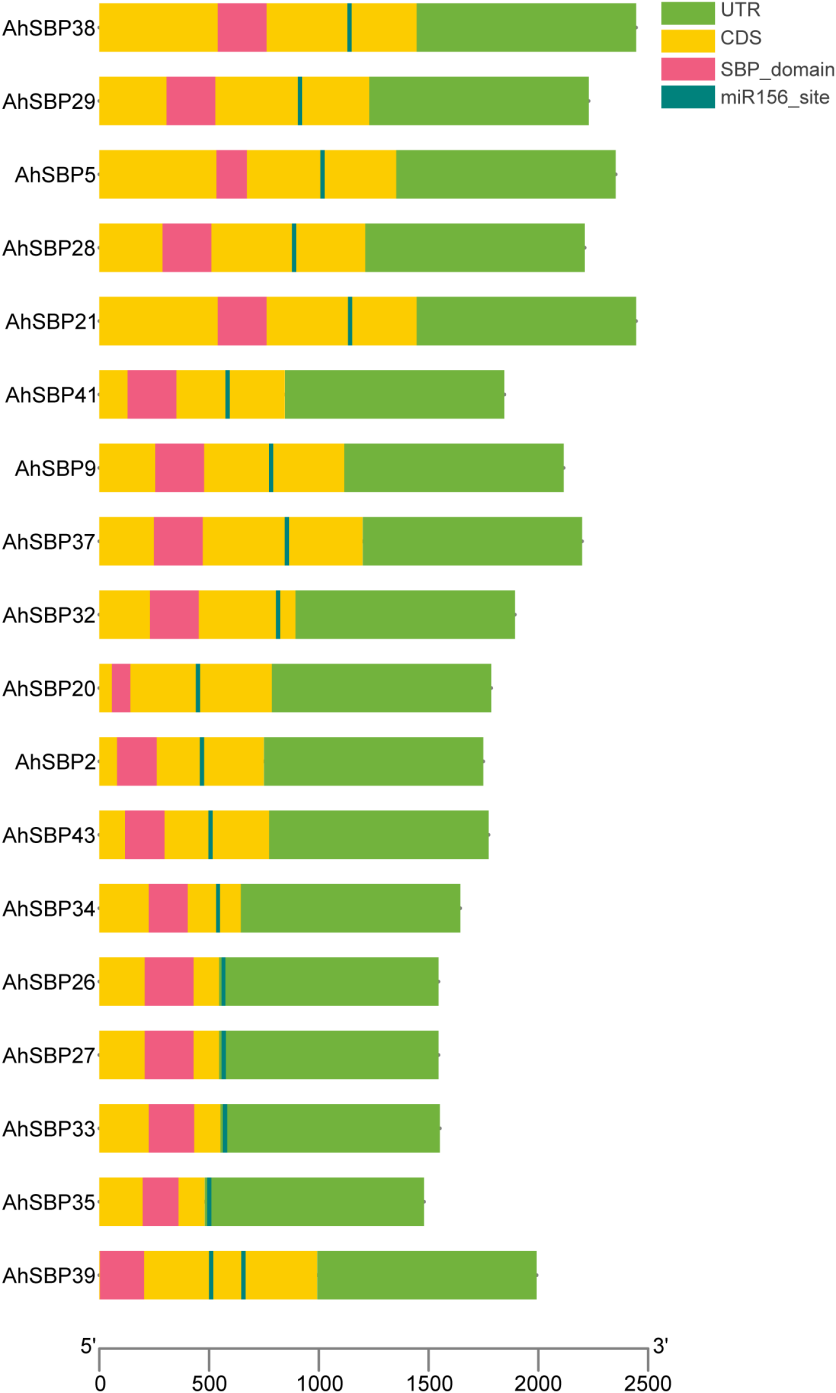
miR156 target site distribution of *AhSBP* genes in sisal. The schematic diagram illustrates the gene architecture and the precise localization of miR156 target sites for 18 *AhSBP* family members. Green boxes represent untranslated regions (UTRs), yellow boxes represent coding sequences (CDS), and pink boxes denote the conserved SBP domain. The teal vertical lines indicate the predicted miR156 target sites. The scale bar at the bottom indicates the nucleotide length (bp) relative to the translation initiation site (ATG), which is designated as position 0. All gene structures are drawn to scale based on the transcript sequences.

The miR156-SBP regulatory hub is a universally conserved module in plants, known to orchestrate the balance between development and environmental stress adaptation. Given its pivotal role in plant plasticity, we hypothesized that a similar regulatory logic might govern the immune landscape of sisal. To elucidate the post-transcriptional regulatory mechanism of *AhSBP* genes by miR156, we performed a comprehensive target site prediction across the *AhSBP* family. Among the identified members, 18 *AhSBP* genes were confirmed to harbor highly conserved miR156 recognition sites (**Figure 7****;****Supplementary Table S7**). Structural analysis revealed that these target sites are predominantly located within the coding sequences (CDS), particularly in the downstream region relative to the SBP domain. Notably, the spatial distribution of miR156 sites exhibited high conservation, in which the target sequence was mainly positioned approximately 100–300 bp downstream of the SBP domain. However, *AhSBP39* presented a unique regulatory architecture, containing two distinct miR156 binding sites, suggesting a potentially more complex or redundant suppression mechanism. Besides, the miR156 target sites of *AhSBP26/27/33/35* genes were located within the 3’UTR region. The presence of these sites within the CDS suggests that miR156 primarily regulates *AhSBP* expression through mRNA cleavage or translational inhibition in sisal.

### Antagonistic expression of the asi-miR156-*AhSBP* module coordinates sisal defense responses to biotic stresses

**Figure 8:**
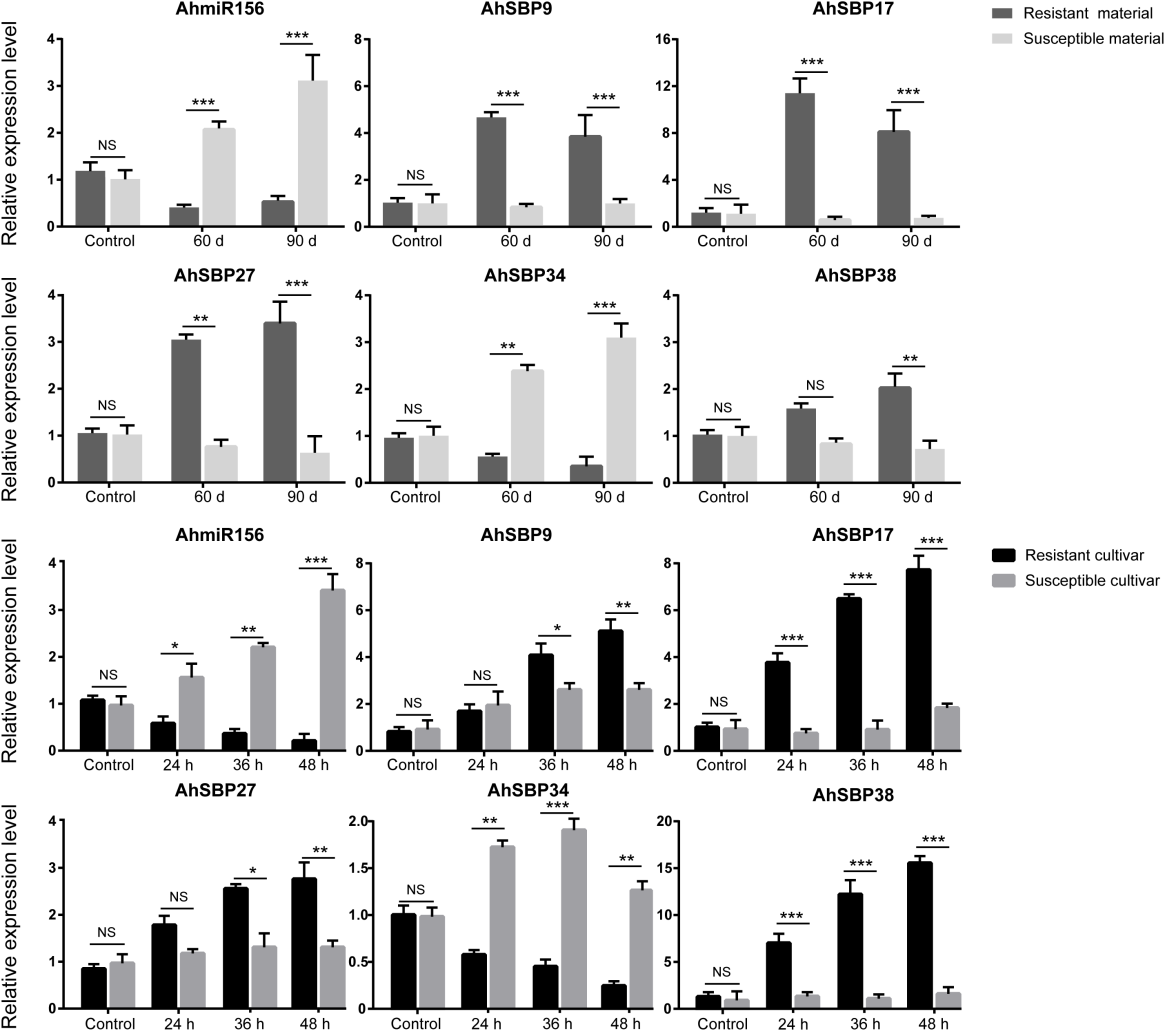
Dynamic expression profiles of asi-miR156 and *AhSBP* genes under pathogen stress. Relative expression levels of asi-miR156 and five representative *AhSBP* genes were determined by RT-qPCR in resistant (R) and susceptible (S) sisal materials. (Top two rows) Expression patterns during purple leaf roll disease at 60 d and 90 d post-inoculation. (Bottom two rows) Expression patterns during zebra stiff leaf disease at 24 h, 36 h, and 48 h post-inoculation. Data represent the mean ± SD of three biological replicates. Statistically significant differences between resistant and susceptible materials at each time point were determined using Student’s t-test (*P < 0.05; **P < 0.01; *P < 0.001). NS represents no significance.

To further elucidate the regulatory role of miR156 in sisal immunity, we selected five candidate genes (*AhSBP9/17/27/34/38*) for detailed RT-qPCR validation across different infection stages of purple leaf roll disease and zebra stripe disease, based on their expression profiles and characteristics as miR156 targets. Our results revealed that the asi-miR156-AhSBP module may serve as a critical regulatory switch. As expected, asi-miR156 was significantly induced in susceptible materials during the late stages of infection (90 d for purple leaf roll disease and 48 h for zebra stripe disease), but was markedly suppressed in resistant materials (**Figure 8**). Correspondingly, four of the selected genes (*AhSBP9*, *AhSBP17*, *AhSBP27*, and *AhSBP38*) showed a strong antagonistic expression pattern relative to asi-miR156. In resistant cultivars, the down-regulation of asi-miR156 coincided with a robust induction of these four genes, particularly *AhSBP17*, which reached a 12-fold increase during purple leaf roll disease infection. Interestingly, while *AhSBP9*, *AhSBP27*, and *AhSBP38* contain canonical miR156 binding sites, *AhSBP17* lacks a predictable site, suggesting that miR156 might regulate its expression through an indirect SBP-mediated cascade or shared immune signaling pathways. In contrast, *AhSBP34*, which was identified as a putative miR156 target with a relaxed expectation value (E = 4.0), displayed a unique expression profile. Unlike the other four members, the expression of *AhSBP34* was higher in susceptible materials and followed a positive correlation with asi-miR156 levels during zebra stripe disease infection. This suggested that AhSBP34 might play a distinct role, possibly acting as a negative regulator of defense or being hijacked by the pathogen to facilitate colonization. Collectively, our integrated analysis uncovers a hierarchical regulatory landscape within the sisal *AhSBP* family, in which miR156 may act as the primary gatekeeper of immune homeostasis. While initial transcriptomic heatmaps highlighted *AhSBP17* and *AhSBP34* as prominent candidates for broad-spectrum pathogen resistance, *AhSBP17* and *AhSBP34* might function at a secondary or downstream level of this immune hierarchy without direct interaction with miR156.

## Discussion

### Comparative genomics highlights the uniqueness and expansion of the *AhSBP* family

The SBP-box gene family is specific to green plants and plays indispensable roles in regulating plant growth, development, and stress responses. In this study, we identified 45 *AhSBP* genes in the *A.sisalana* hybrid 11648 genome. This number is significantly higher than those reported in model species such as *A.thaliana* (17 members) and *O.sativa* (19 members) (Yang et al., 2007), and loquat (28 members) (Song et al., 2023), even approaches that of the polyploid species wheat (48 members) (Li et al., 2020). This substantial increase provides strong evidence for a lineage-specific expansion in sisal, suggesting that these genes have undergone adaptive evolution to meet the unique challenges faced by this monocot. The physicochemical heterogeneity of AhSBP proteins, particularly the broad range of molecular weights and pI values, mirrors findings in other monocots including *Euphorbiaceae* (Li et al., 2019), indicating that structural diversification is a common evolutionary strategy to accommodate diverse biological functions. AhSBP proteins could be categorized into short and long types, a structure also found in other species. These two types likely play opposite roles: long AhSBPs act as activators, while short ones act as repressors or interaction partners. This balanced system is common in flowering plants and is essential for precisely regulating how they grow (Preston and Hileman, 2013). We also discovered how microRNAs help fine-tune the *AhSBP* family. In sisal, asi-miR156 targets 18 different *AhSBP* genes to manage their activity. This is a relatively small portion of the family compared to that in oats (16 *AsSPL*s/28 *AsSPLs*, Mehtab-Singh et al., 2024) and barley (7 *HvSPLs*/*17 HvSPLs*, Tripathi et al., 2018). This lower percentage suggests that miR156 has a more selective role in controlling how sisal grows and responds to stress. Establishing this comprehensive dataset fills a critical gap in our understanding of transcription factors in CAM (Crassulacean Acid Metabolism) plants and fiber crops, providing a reference framework for future comparative studies in the *Asparagaceae* family.

### Evolutionary trajectory driven by synergistic duplication events

Unraveling the evolutionary history of gene families is pivotal for understanding their functional adaptation. Our phylogenetic and syntenic analyses reveal that the expansion of the *AhSBP* family was driven by a synergistic interplay of segmental and tandem duplications, with segmental duplication accounting for over 70% of the events. This pattern contrasts with species such as oil palm, where tandem duplication is the primary driver (Zhou and Yarra, 2023), but aligns with the evolutionary history of beet (Xue et al., 2024). Besides, We identified two major ‘expansion hubs’ on LG01 and LG03. Specifically, the *AhSBP14* network on LG03 extends across four different chromosomes (LG02, LG14, LG20, and LG28). This widespread retention of gene copies provides strong evidence for ancient whole-genome duplication (WGD) events in sisal. These duplicated genes likely developed specialized roles through subfunctionalization, ultimately enhancing sisal’s ability to adapt to its environment. Furthermore, the monocot-specific clustering of *AhSBP* genes with *O.sativa* orthologs (Including Group I and III) underscores their deep evolutionary conservation. Notably, the single clustering of eight sisal genes in Group II indicates a recent expansion specific to the *A.sisalana* lineage. This recent expansion may have been driven by adaptive selection in response to sisal’s unique environmental challenges, such as fiber development or adaptation to arid tropical environments, highlighting the dynamic nature of the *AhSBP* genome. In summary, the AhSBP family represents a balance between ancient stability and recent innovation. This combination likely provides the genetic flexibility needed for sisal to thrive in its unique environment.

### Multi-layered regulatory networks of *AhSBP* genes in sisal defense

Deciphering the multi-layered regulatory networks of the AhSBP family is fundamental to understanding how sisal responds to biotic stress. Our analysis suggests that the roles of *AhSBP* genes are first encoded in their unique promoter architectures (**Figure 5**). We found that *AhSBP* promoters are heavily enriched with light-responsive and hormone-responsive motifs, such as the SA-related TCA-elements in *AhSBP9* and *AhSBP38*. This structural arrangement is consistent with emerging models in plant immunity, where light signaling and hormone crosstalk (particularly JA/ABA) are tightly integrated to modulate defense responses (Ballaré, 2014; Pieterse et al., 2012). With these diverse elements, *AhSBP* genes are ‘ready’ to sense environmental and internal signals, helping them coordinate sisal’s defense strategy.

These regulatory potentials are clearly reflected in the dynamic expression patterns observed in our transcriptomic analysis (**Figure 6**). The enrichment of specific motifs appears to drive the correspondingly transcriptional signatures under pathogen stress. For instance, *AhSBP17* exhibited sustained upregulation in resistant cultivars across both chronic and acute infections, which possesses a high density of SAR-related elements. *AhSBP34* was consistently induced in susceptible materials, which is rich in ABA-responsive motifs but lacks SA/JA clusters. Given that pathogens often hijack ABA signaling to suppress plant immunity, the promoter-driven induction of *AhSBP34* in susceptible lines may mark it as a potential negative regulator.

While the promoter and transcriptome reveal the main expression patterns, the asi-miR156-AhSBP module provides a second, post-transcriptional layer of precision. We identified 18 *AhSBP* genes as targets of miR156, with most binding sites located within the coding sequences (**Figure 7**). RT-qPCR validation confirmed that this module acts as a critical regulatory switch during infection (**Figure 8**). In susceptible plants, the significant induction of asi-miR156 correlates with the suppression of key targets like *AhSBP9, AhSBP27*, and *AhSBP38*. This antagonistic relationship reinforces the role of the miR156-AhSBP module as a primary gatekeeper of immune homeostasis, probably acting as a ‘brake’ on the hormone-mediated defense signals predicted by our promoter analysis.

Interestingly, our combined analysis shows a clear division of labor among *AhSBP* genes. Some act as primary controllers, while others may function further downstream. While miR156 directly manages primary targets like *AhSBP9* and *AhSBP38*, our findings suggest that candidates like *AhSBP17* and *AhSBP34* function differently. *AhSBP17* lacks a canonical miR156 binding site yet still shows an antagonistic expression pattern, suggesting it may be regulated through an indirect SBP-mediated cascade. Meanwhile, the positive correlation between *AhSBP34* and miR156 levels distinguishes its role from the primary defensive SBPs. Overall, these findings show that miR156 directly targets only a few specific genes. Meanwhile, others like *AhSBP17* and *AhSBP34* likely act as downstream helpers that strengthen sisal’s complex defense system. Further functional validation is expected to confirm the precise roles of these genes in sisal’s intricate defense mechanisms.

## Conclusion

In summary, this study provides the first systematic characterization of the *SBP-box* gene family in sisal, elucidating its genomic organization, evolutionary history, and functional implications in disease resistance. We demonstrated that the expansion of the *AhSBP* family was shaped by ancient polyploidization and recent local duplications, resulting in a structurally diverse repertoire of transcription factors. By combining promoter analysis, transcriptomic data, miR156 targeting and qPCR, we uncovered the essential role of *AhSBP* genes in controlling sisal’s defense responses. These findings not only advance our understanding of transcriptional regulation in fiber crops but also provide potential genetic resources for breeding superior, disease-resistant varieties to sustain the global sisal industry.

## Data Availability

The datasets presented in this study can be found in online repositories. The names of the repository/repositories and accession number(s) can be found in the article/**Supplementary Material**.
